# Automatic transformation of Kazakh text into sign language glosses using multilingual transformer-based models

**DOI:** 10.3389/frai.2026.1867966

**Published:** 2026-08-07

**Authors:** Nurzada Amangeldy, Aigerim Yerimbetova, Nazira Tursynova, Nazerke Gazizova, Nazgul Dospol, Arif Orymbetov

**Affiliations:** 1 Institute of Information and Computational Technologies of the Committee of Science of the Ministry of Science and Higher Education of the Republic of Kazakhstan, Almaty, Kazakhstan; 2 Faculty of Information Technologies, L. N. Gumilyov Eurasian National University, Astana, Kazakhstan; 3 SignBridge LLP, Astana, Kazakhstan; 4 META University, Almaty, Kazakhstan; 5 Faculty of Applied Sciences, Esil University, Astana, Kazakhstan

**Keywords:** gloss annotation, Kazakh sign language, low-resource language, mT5, multilingual transformer, sign language processing, text-to-gloss translation

## Abstract

This study investigates the automatic transformation of Kazakh text into sign language glosses (Text-to-Gloss) using multilingual transformer-based models with emphasis on preserving morphological structure in a low-resource agglutinative language framework. Given the scarcity of high-quality intermediate representations for Kazakh Sign Language, a methodology for corpus formation was developed, resulting in a specialized dataset of 11 190 unique text−gloss pairs sourced from educational materials. To assess the effectiveness of transfer learning under low-resource constraints, we fine-tuned mT5-small and mT5-base models on this dataset. Experimental results indicate that the mT5-base architecture consistently outperforms the smaller variant across all metrics, achieving a 2.61-point improvement in BLEU (87.51 → 90.12) and a 2.47-point increase in ROUGE-1 F1 (89.61% → 92.08%), alongside gains in exact match (74.96% → 80.68%) and chrF (91.6 → 94.1). Statistical significance testing (*p* < 0.001) confirms the stability of this improvement. Error analysis reveals that larger model capacity particularly improves morphological handling and token coverage, which is critical for agglutinative languages, while maintaining semantic accuracy. These findings demonstrate that pretrained multilingual models effectively address TTG transformation for low-resource agglutinative languages through transfer learning, with practical applications for Kazakh Sign Language assistive systems and education.

## 1 Introduction

Despite the rapid advancements in artificial intelligence technologies and large language models (LLMs), sign languages are still poorly integrated into modern digital systems. The problem is not so much the limitations of individual components, such as recognition or gesture generation, but rather the lack of a reliable intermediate representation that enables correct transformation between linear text and spatial−visual forms. In existing approaches, this gap leads to a loss of meaning, distortion of grammatical connections, and a decrease in the comprehensibility of the results, which is especially important for morphologically complex and low-resource languages.

Current research shows a large imbalance in digital support for sign languages. There are about 119 sign languages in the world, but few have enough data and technological infrastructure to integrate into automatic translation and avatar generation systems (Sultan et al., 2022; Martínez-Sánchez et al., 2023). It is estimated that about 38 languages have structured lexicons with more than 1,000 annotated glosses, and the number of languages with proven avatar systems does not exceed 20. Resources are concentrated around a limited number of well-endowed languages, such as American Sign Language (ASL), British Sign Language (BSL), and German Sign Language (DGS). The largest standardized datasets, such as RWTH-PHOENIX, ASLLVD, and MX-ITESO-100, have been created for them, which serve as benchmarks for the development and evaluation of recognition and gesture generation models.

The glosses produced by a text-to-gloss model such as the one proposed here are not an end product in themselves, but an intermediate representation intended for downstream avatar-based rendering pipelines. As a concrete example of this pipeline, our companion work presents a bilingual (Kazakh−Russian) sign language avatar that consumes gloss sequences and maps them to skeletal and facial animation, validated through expert evaluation by certified interpreters (Amangeldy et al., 2026). This illustrates the practical role of the TTG stage addressed in the present study within a complete text-to-sign-language communication pipeline.

In contrast, many sign languages common in Africa, South America, and some regions of Asia remain poorly represented in scientific and technological developments. For these languages, there are no standardized parallel corpora and consistent glossing systems, which greatly limit the portability of models and reduce the effectiveness of learning. As a result, there is a structural inequality: modern methods show high productivity only for languages with rich resources.

The key approach to solving this problem is to consider the conversion of text into glosses (Text-to-Gloss, TTG) as the central element of the computational process, which provides structural alignment between different modalities. Formalization of this stage using standardized corpus resources and transformer-based models allows for capturing the linguistic features of the language and reducing information loss during the transition to sign representation. However, despite the development of transformer-based architectures and transfer learning methods, the TTG problem remains insufficiently studied for low-resource languages.

Attempts to bridge this gap are related to the development of interlingual transfer learning approaches. Recent research suggests that models trained in resource-rich languages such as ASL or GSL can be adapted to related or less-represented languages, including International Sign Language (ISL-Int), using data augmentation techniques (Wolfe et al., 2022; Arib et al., 2025). This achieves up to 90% preservation of semantic accuracy, which demonstrates the potential of multilingual modeling to expand coverage. However, such approaches do not eliminate the main dependence on the quality and structure of the source data and do not solve the problem of the lack of unified annotation standards.

Thus, despite the increasing availability of data for low-resource sign languages, the development of this area remains uneven. Further progress requires standardized annotation methods, multilingual learning platforms, and active collaboration with deaf communities to ensure linguistic authenticity and fair technological representation (Sultan et al., 2022; Wolfe et al., 2022).

In this regard, the purpose of this study is to develop and experimentally validate an approach to addressing the problem of transformation between text and spatial-visual modalities, based on the use of TTG as a central component of the computational pipeline, including the creation of a corpus, the formation of a dataset, and the training of transformer models in a low-resource scenario.

## 2 Literature review

The architecture of modern computational pipelines for sign language processing is classified into three main directions. These include recognition (Sign Language Recognition, SLR), machine translation (Sign Language Translation, SLT), which includes a critical step of transforming natural text into glosses, and automatic gesture generation (Sign Language Production, SLP).

In the SLR direction, two feature extraction approaches are predominant, based on computer vision (CV) and wearable sensors. The use of inertial measurement units (IMUs), such as smart rings (Li et al., 2022, 2023; Zhu et al., 2023), addresses the problem of visual occlusion, but creates strong dependence on specific hardware, which limits the ability to assemble large-scale training datasets. CV methods, including audiovisual speech recognition models (Kumar et al., 2022) and multilayer character-by-character articulation analysis (Ali et al., 2022; Amangeldy et al., 2025), can achieve a word error rate (WER) of 6.59%. To improve the reliability of recognition of isolated gestures, late fusion mechanisms in ensemble networks are used (Hrúz et al., 2022), but this leads to a significant increase in computational load at the inference stage.

The SLP direction is characterized by a similar trade-off between visualization quality and computational cost. Deterministic 3D avatars (Kipp et al., 2011) and monocular video-based kinematic translators (Zuo et al., 2025) provide low generation latency but have limited naturalness. The introduction of generative diffusion models (Tang et al., 2025) allows for maximizing the quality of frame synthesis (according to the Fréchet Inception Distance (FID) metric) and motion continuity. However, the computational complexity of diffusion sampling, along with the latency of multilingual generative architectures based on LLMs (Fang et al., 2025), significantly limits their application in simultaneous interpretation systems. Consequently, the hardware limitations of the SLR domain and the computational redundancy of SLP models indicate that the core of the semantic coherence of the entire pipeline remains the intermediate stage of TTG translation. It is at this stage that the main focus of linguistic and algorithmic modeling should be shifted to ensure translation accuracy.

The fundamental problem of TTG translation is the conceptual delimitation of nominative units when changing the modality from the auditory-linear to the spatial-visual. The theoretical description of the differential parameters of a word and a word combination (Diachok et al., 2022) demonstrates the inadvisability of direct verbal comparison between text and sign equivalents. For morphologically complex languages, this problem is aggravated by a significant shortage of annotated parallel corpora. The effectiveness of neural network translation is largely determined by the volume and depth of annotation. For example, maintaining translation accuracy for the Oran dialect of Arabic required the creation of the MADOran corpus containing 30,919 words with detailed manual annotation of root morphemes and affixes (Sawalha et al., 2025).

In the context of Kazakh Sign Language, the creation of parallel corpora of comparable quality is still at an early stage (Yerimbetova et al., 2025). The lack of detailed alignment of text−gloss pairs prevents effective training of deep neural networks. The importance of structural annotation for low-resource languages is statistically confirmed in a diachronic study of machine translation quality for Urdu (Shah et al., 2024). The authors state that, despite continuous updates to Google Translate architectures, the level of syntactic errors in translations remains high (about 50%). The analysis showed that without interlinear glossing methods that explicitly specify core semantic units, probabilistic models tend to insufficiently account for spatial-grammatical relationships. This implies that for agglutinative languages such as Kazakh, direct projection of affix chains onto glosses without prior morphological parsing can lead to significant distortions of the syntactic and semantic structure.

The historical evolution of TTG systems demonstrates the transition from deterministic expert systems to deep learning architectures (Shahin and Ismail, 2024). Early approaches based on rigid syntactic rules provided high grammatical accuracy, but their scalability was critically limited. In an attempt to overcome this barrier, methods of algorithmic transcription simulation have been proposed, such as neural editing programs (Li et al., 2022), where the process of gloss generation is reduced to predicting discrete operations (copying, deleting, and adding). This approach demonstrates improved performance in terms of the ROUGE-L metric over standard sequence-to-sequence (seq2seq) architectures, but a predefined action space limits the modeling of complex concurrent morphology.

The modern standard in TTG tasks is the use of transformer-based architectures and transfer learning methods. The use of ULMFiT models to eliminate lexical ambiguity (Patil et al., 2022) and advanced BERT architectures with multi-head attention (Waghmare and Deshpande, 2024) enables more efficient extraction of contextual dependencies. To compensate for the significant deficit of annotated pairs, self-supervised learning methods are actively introduced. In particular, Dang et al. (2023) uses the reverse translation task as a preliminary step for Korean Sign Language, which provides a 6.22% performance gain over the base BERT model. The requirements for real-time systems development stimulate the use of similar architectures. For example, the Arabic Sign Language translator (Mosa et al., 2023) relies on BERT embeddings in conjunction with attention mechanisms, but the authors required the manual creation of a gloss dataset consisting of 187 pairs to achieve acceptable accuracy. However, empirical evidence suggests structural limitations of the application of deep transfer learning in this subject area. The study of transferring “frozen” pre-trained representations (De Coster and Dambre, 2022) shows that the use of purely text encoders results in only a marginal increase in the BLEU-4 metric (1.5–2.0 points). Attention mechanisms trained on linear text are not sufficiently effective for accurate modeling in a multidimensional space of sign parameters.

The desire to minimize the loss of information at the glossless stage led to the development of the Gloss-Free SLT paradigm. The use of normalized skeletal points (Kim and Baek, 2023) and the integration of spatial-dynamic features with LLMs (Hwang et al., 2025) allow translation to be generated directly from visual representations. Despite achieving a SacreBLEU score of 18–20 on the How2Sign dataset, glossless methods are associated with a noticeable decrease in accuracy in certain aspects. In particular, the absence of an explicit intermediate representation leads to the degradation of stimulus morphology reproduction and fine motor skills (fingerspelling). Moreover, generation based on LLMs is subject to “hallucinations,” which is unacceptable in precision translation. The need for explicit simulation (simultaneous expression of manual and non-manual components) is mathematically justified in translation into Indian Sign Language (ISL) (Bhagwat et al., 2024), which calls into question the feasibility of completely eliminating glosses for morphologically complex languages (see Table 1).

**Table 1:** Comparative analysis of approaches to translation into sign languages.

Approach	Key work	Quality metrics	Benefits	Limitations
Neural editing (Seq2Seq)	(Li et al., 2022)	+2–4% ROUGE-L	Formalizes the glossification process through editing operations	Limited action space; weak simulation of simultaneous morphology
Transformer-based (BERT/ULMFiT)	(Patil et al., 2022; Waghmare and Deshpande, 2024)	BLEU-4 < 25	Improved context extraction; effective transfer training	High data volume dependency; limited adaptation to agglutinative morphology
Self-supervised learning	(Dang et al., 2023)	+6.22% over baseline	Reduces reliance on annotated data	Sensitivity to noise and pseudo-marking quality
Frozen pre-trained transformers	(De Coster and Dambre, 2022)	+1.5–2.0 BLEU-4	Fast adaptation without full training	Limited projection of textual attention on sign structure
Gloss-free (keypoints)	(Kim and Baek, 2023)	−3% to 5% ER	Simplification of the pipeline; reduction of recognition errors	Loss of explicit morphological representation
LLM Integration (SpaMo)	(Hwang et al., 2025)	SacreBLEU ~18–20	Unification of visual and linguistic representations	High computational cost; risk of semantic deviations

Existing methods of sign language processing demonstrate significant progress in individual components, but do not provide a comprehensive solution to the problem of intermodal transformation. The analysis reveals that the limitations of recognition systems are due to their dependence on sensory data and vulnerability to occlusion, while gesture generation methods face a trade-off between quality and computational complexity. In such conditions, the main responsibility for ensuring the semantic coherence of the pipeline is assigned to the intermediate stage of transforming the text into glosses.

Existing approaches to transforming text into glosses are either limited by rigid rules or depend on the availability of annotated data and are not sufficiently adapted to morphologically complex languages. Alternative glossless methods, despite the simplification of the architecture, lead to the loss of structural information and a decrease in the accuracy of simultaneous morphology reproduction. This indicates that the rejection of the intermediate representation does not eliminate the problem but only redistributes it.

Thus, the key task is to develop coordinated corpus resources and models that can provide a structurally correct transformation of text into glosses, while accounting for the linguistic features of the language. This problem is especially relevant for low-resource and agglutinative languages, for which there are no standardized glossing methods and sufficient training data.

## 3 Materials and methods

**Figure 1:**
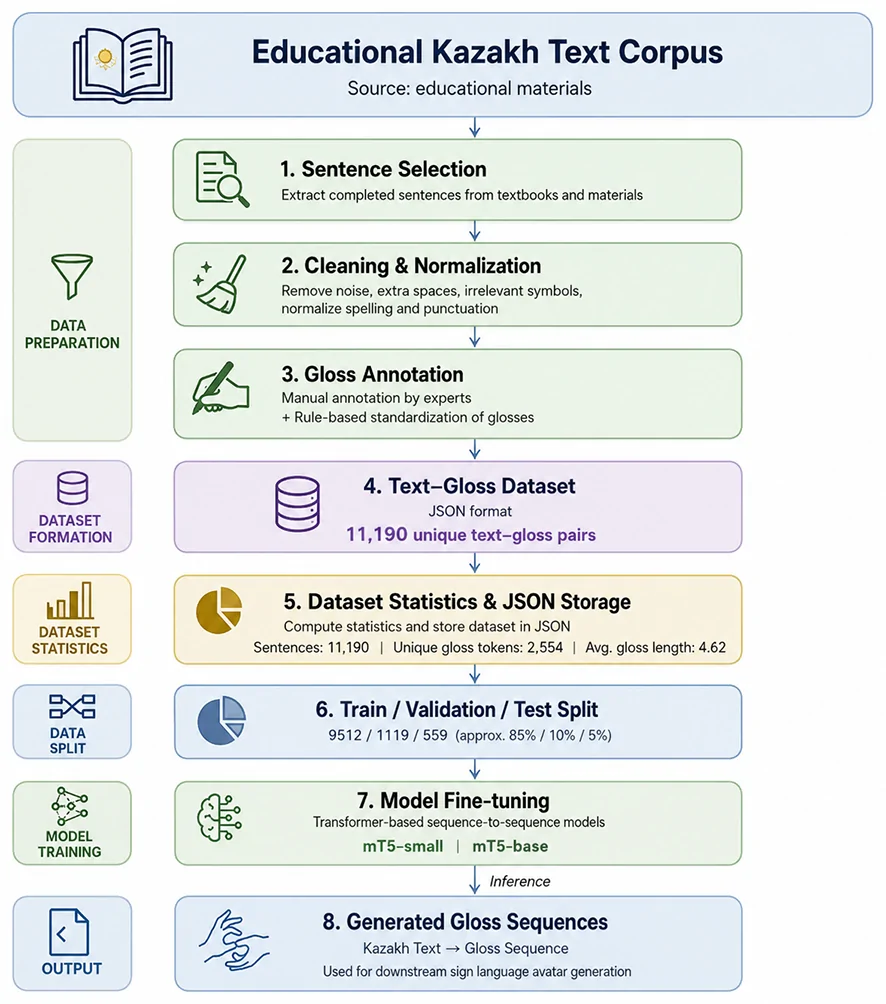
The general scheme of corpus preparation, dataset formation, and TTG model training.

Within the framework of this study, a methodology for forming a corpus and a dataset for the task of TTG transformation was developed, as well as experimental training of models based on the obtained data. The overall process includes the stages of selecting text material, its preparation, annotation, formation of text–gloss pairs, and their subsequent use for training and model validation. A general outline of the proposed approach is shown in Figure 1.

After the general description of the methodology, the individual components of the process are discussed in detail, including the characteristics of the corpus, the procedures for data preparation, and the features of dataset formation.

### 3.1 Corpus characteristics

In this study, a dataset of pairs consisting of “a sentence in the Kazakh language–a sequence of glosses in Kazakh Sign Language” was used to solve the problem of transforming written natural text into an intermediate formalized representation suitable for subsequent machine processing. Within the framework of the TTG problem, the initial object is a sentence or a completed statement in the Kazakh language, and the target representation is a sequence of glosses that represents the semantic and structural units used in subsequent computational procedures. This representation is considered not the final form of sign language expression, but rather an intermediate level of description used both for training models of automatic TTG conversion and for subsequent modules that generate sign representations.

The source text material is based on school educational resources in the Kazakh language. The corpus includes sentences and fragments reflecting typical forms of educational communication: narrative statements, definitions, explanatory constructions, questions, task elements, and subject-specific formulations. Thematically, the texts cover various school disciplines, including natural science and general education areas. The choice of this type of sources is due to the normative language design, relative lexical and grammatical stability, thematic organization, and didactic orientation of school materials. These properties make such texts a methodologically grounded basis for building a training-oriented TTG corpus, in which the predictability of syntactic structures, the repeatability of terminology, and the presence of semantically transparent statements play an important role.

The use of school texts allows the corpus to be oriented not only toward computational tasks but also toward practical application scenarios. Educational sentences are typically complete and interpretable statements related to the explanation of concepts, the description of the properties of objects, the establishment of causal relationships, questioning, and the formulation of tasks. For the TTG problem, this is important because such texts provide a variety of syntactic and semantic constructions while maintaining semantic clarity.

### 3.2 Data preparation

The corpus was prepared through several interrelated stages: selection of text material, preprocessing, normalization of the record, and subsequent annotation in gloss form. At the selection stage, fragments were extracted from the original teaching materials, which are independent and meaningfully completed statements. Preference was given to sentences that retain interpretability outside the broader context of the page or task and allow representation in the form of a separate text–gloss pair. This approach reduces the dependence of the annotation on the external context and increases the suitability of the data unit for automated processing.

After the selection, the text was cleaned and unified. Elements that impede direct use in a paired format were excluded from the source materials, including technical formatting artifacts, excessive gaps, heterogeneity of punctuation, and other features that are not related to the language content of the statement. The text was brought to a uniform format, ensuring compatibility of records within the corpus. The normalization was aimed at minimizing variability that does not serve an independent semantic distinguishing function within the framework of the problem under consideration, as well as at preparing initial sentences for sequential annotation.

The transition from a written Kazakh sentence to a gloss record was considered as an independent annotation process in which the target sequence does not reproduce verbatim the structure of the original sentence. Glosses were used as a formalized intermediate representation, based on features of sign language description and the requirements of machine processing. Thus, the annotation did not imply a mechanical word-by-word correspondence, but rather the structuring of the original content into a sequence of units suitable for subsequent use in TTG modeling.

**Figure 2:**
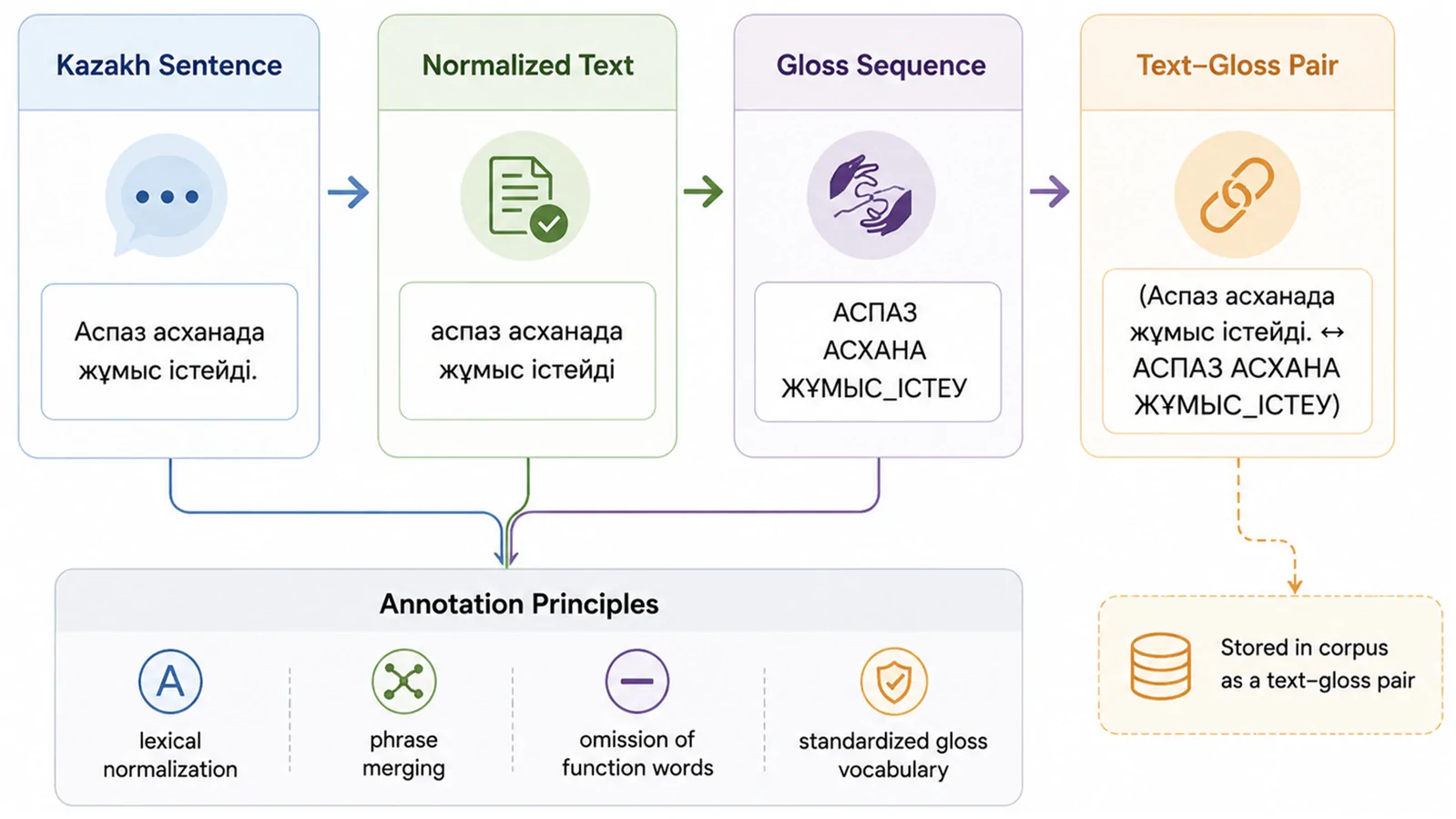
Transformation of Kazakh text into gloss representation and annotation principles in the TTG framework.

In the formation of the gloss sequence, the principles of lexical normalization and standardization of representation were applied. For the same content, standardized basic lexical units were selected, and representation variants were reduced to a single form within the corpus. If necessary, elements of written text that are not essential to the formalized gloss presentation, including functional elements or redundant repetitions, were eliminated if their retention did not increase the consistency of the annotation. In cases where the written structure of the sentence did not correspond to the intended organization of the gloss sequence, simplification or restructuring of the order of units was allowed while maintaining the basic meaning. This approach is fundamentally important for the TTG resource, since written Kazakh text and gloss representations of sign language do not exhibit strict linear isomorphism (see Figure 2). In addition to the example shown in Figure 2, Table 2 presents further illustrative text–gloss pairs covering annotation phenomena not previously demonstrated: explicit negation marking, categorization of named entities via generic placeholder tags, and the treatment of semantically empty function words using the dedicated *O* tag.

**Table 2:** Additional text–gloss examples illustrating annotation conventions.

Kazakh text	Gloss
Бүгін жаңбыр жауып тұр. *It is raining today*.	Бүгін | жаңбыр *today | rain*
Егер уақытб олса, келемін. *If I have time, I will come*.	Егер | уақыт | бар | келу *if | time | have | come*
Бала билей алады. *The child can dance*.	Бала | билеу | істей_алу *child | dance | can/do_able*
Aспаз асханада жұмыс істейді. *The chef works in the kitchen*.	Aспаз | асхана | жұмыс_істеу *chef | kitchen | work*
Жер күнді айналады. *The Earth revolves around the Sun*.	Жер | күн_sun | айналу *earth | sun | revolve*

These examples illustrate several transformation rules used during gloss generation. First, auxiliary verbs that do not contribute independent semantic information are omitted (e.g., тұр in the first sentence). Second, context-dependent lexical normalization is applied: the auxiliary verb алады is converted to the gloss істей_алу when it expresses the modal meaning *can/be able to*, rather than its lexical meaning *take* (third example). Third, multi-word expressions that correspond to a single sign are merged into one gloss token (e.g., жұмыс істейді → жұмыс_істеу, fourth example). Finally, semantic tags are added to resolve lexical ambiguity when a Kazakh word has multiple meanings, such as күн, which may denote either **sun** (күн_sun) or **day** (күн_day); therefore, the appropriate English semantic tag is appended to the gloss (fifth example).

Particular attention was paid to the uniform presentation of numerals, terminological units, and repetitive semantic constructions. Standardization of tokens reduced the variability of target sequences not related to content differences, which increased the suitability of the corpus for algorithmic learning. Annotation performed a dual function: translating a written statement into a structured form oriented toward sign representation and forming a consistent machine-processable resource with a uniform internal organization of each pair.

Thus, data preparation ensured the consistency of annotation and the reproducibility of corpus formation. The general scheme of TTG dataset formation includes the following stages: source educational texts → selection of sentences → cleaning and normalization → annotation into gloss sequences → formation of text–gloss pairs → corpus storage → use in model training.

### 3.3 Dataset representation and statistics

The dataset is implemented in JSON format, where each entry contains a pair of the original Kazakh sentence and its corresponding gloss sequence. This form of storage provides a compact and unambiguous representation of data that is convenient for software loading, filtering, verification, and conversion. The JSON structure allows the dataset to be used in standard computational pipelines without the need for intermediate manual conversion, which is especially important for text preprocessing, tokenization, dictionary formation, batch preparation, and automated sampling (Wei et al., 2025).

Choosing JSON as a storage format is also associated with the possibility of further scaling the dataset. When the overall data structure is preserved, new pairs, thematic sections, and additional levels of annotation can be added to the dataset if required at the next stages of the study. This structure does not limit dataset expansion of the dataset and allows its adaptation to new experimental scenarios, including changes in data partitioning, the integration of auxiliary metadata and subsequent processing modules. Thus, the dataset is a reproducible and extensible basis for the development, testing, and comparative analysis of systems for automatic conversion of Kazakh text into glosses of Kazakh Sign Language.

**Figure 3:**
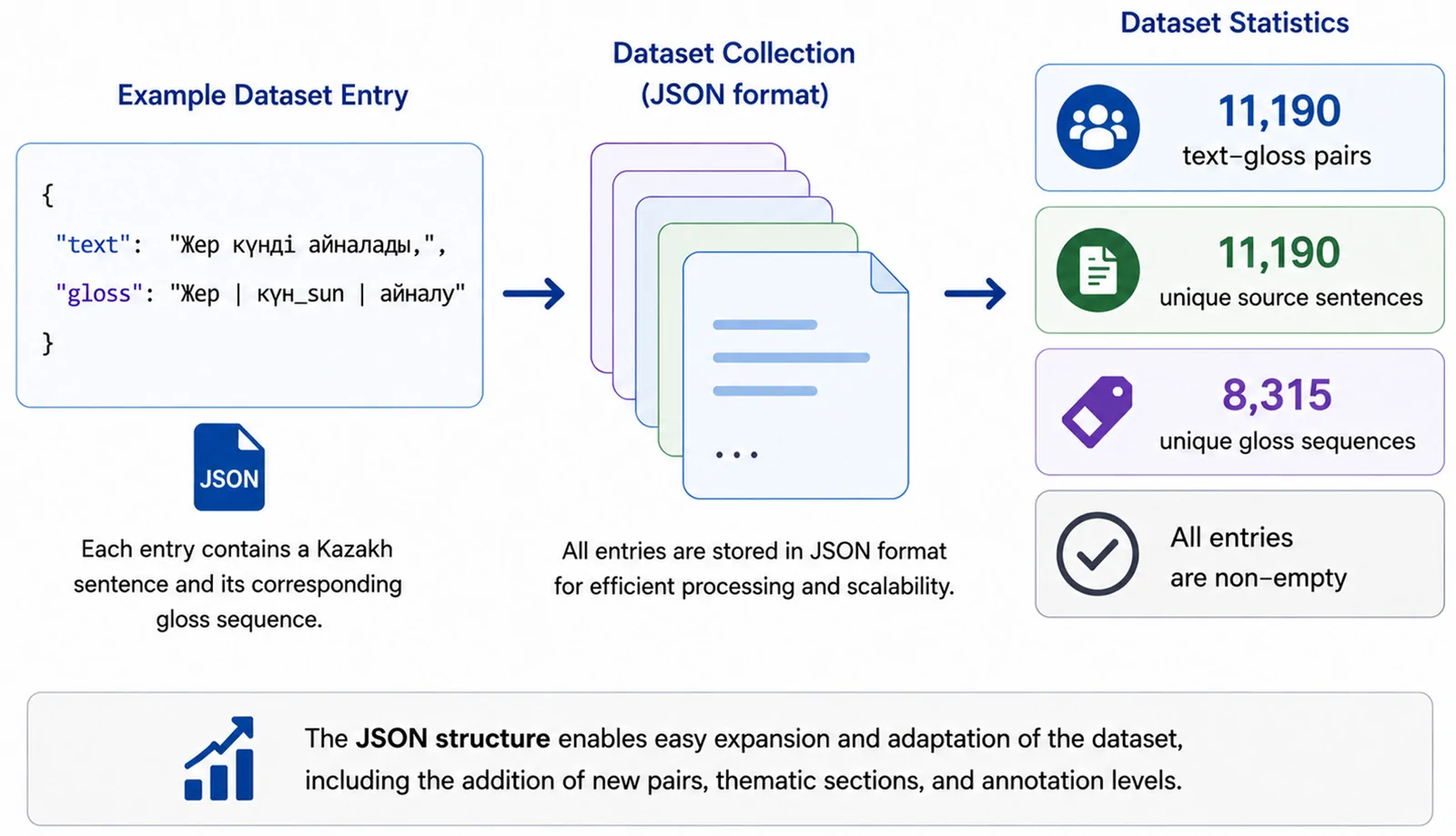
Structure and distribution of text–gloss pairs in the dataset.

The dataset includes 11,190 text–gloss pairs, with all entries being non-empty and unique on the source side. The number of unique target sequences is 8,315, indicating that multiple source sentences may correspond to the same gloss representation. This feature reflects the degree of normalization of the target space and is acceptable within the TTG task (see Figure 3).

**Figure 4:**
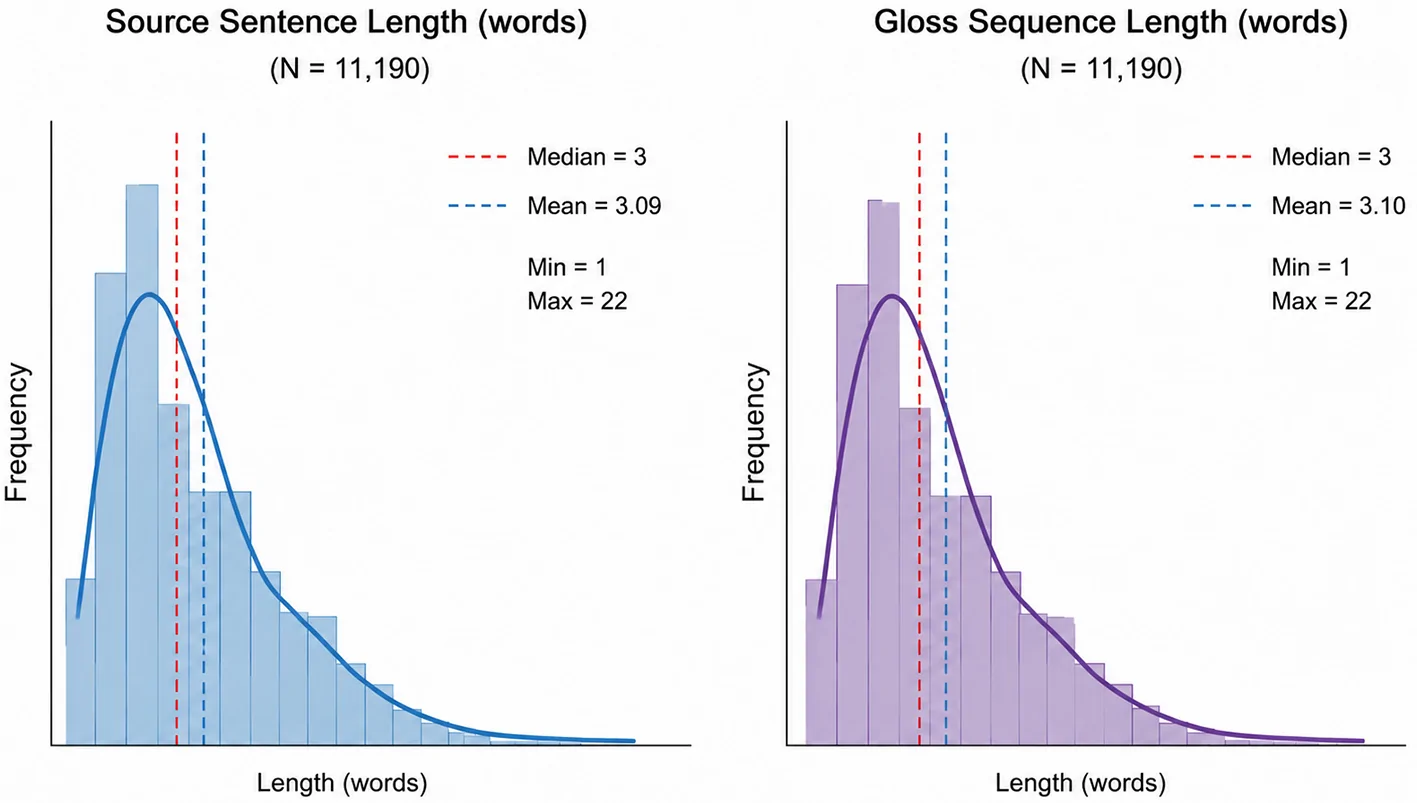
Length distribution of source sentences and gloss sequences.

From a quantitative point of view, the dataset is characterized by variability in length on both the source and target gloss sides (Fayyazsanavi et al., 2024). The corpus contains 11,190 aligned source–gloss pairs. The length of the source sentences ranges from 4 to 186 characters, with a median of 19 characters. On the target side, gloss sequences range from 1 to 171 characters, with a median of 17 characters. When measured in words, the source sentences range from 1 to 22 words, with a median length of 3 words. Similarly, gloss sequences range from 1 to 22 words, with a median length of 3 words (see Figure 4).

The obtained statistics show that the dataset contains both short and relatively longer sentence–gloss pairs, providing varying levels of complexity for text-to-gloss generation.

### 3.4 Experiments

The experimental study was carried out on the basis of the dataset formed within the framework of the present study and based on the described corpus, including 11,190 unique text–gloss pairs. To ensure reproducibility and reduce the risk of systematic sampling error, the data separation process was fully automated.

The dataset was divided into training and validation sets using the split_pairs() function at the val_ratio = 0.1. Prior to the separation, random shuffling was performed, which ensured a more uniform distribution of syntactic constructs of varying complexity between subsets.

As a result, the following dataset sizes were obtained: training set (train): 9,512 pairs, validation set (validation): 1,119 pairs, test set (test): 559 pairs. The selected ratio provides sufficient data for training while maintaining the representativeness of the validation assessment (Chen et al., 2022).

In this experiment, two pre-trained models were used: google/mt5-small and google/mt5-base (Xue et al., 2021). Training was performed iteratively with the calculation of the loss function on the validation set (eval_loss) after each epoch.

The optimal model checkpoint was selected based on the lowest validation loss (eval_loss) observed on the validation set (Kessels et al., 2025). To prevent overfitting, an early stopping mechanism was used with the parameter patience = 3, in which training was stopped if there was no improvement on the validation set for three consecutive epochs.

In the process of training, differences in the convergence dynamics were observed. For the mt5-small model, the early stopping was triggered in the 16th epoch (best checkpoint at epoch 11), while the mt5-base model achieved its best performance in the 35th epoch (out of 40 epochs trained before early stopping).

**Figure 5:**
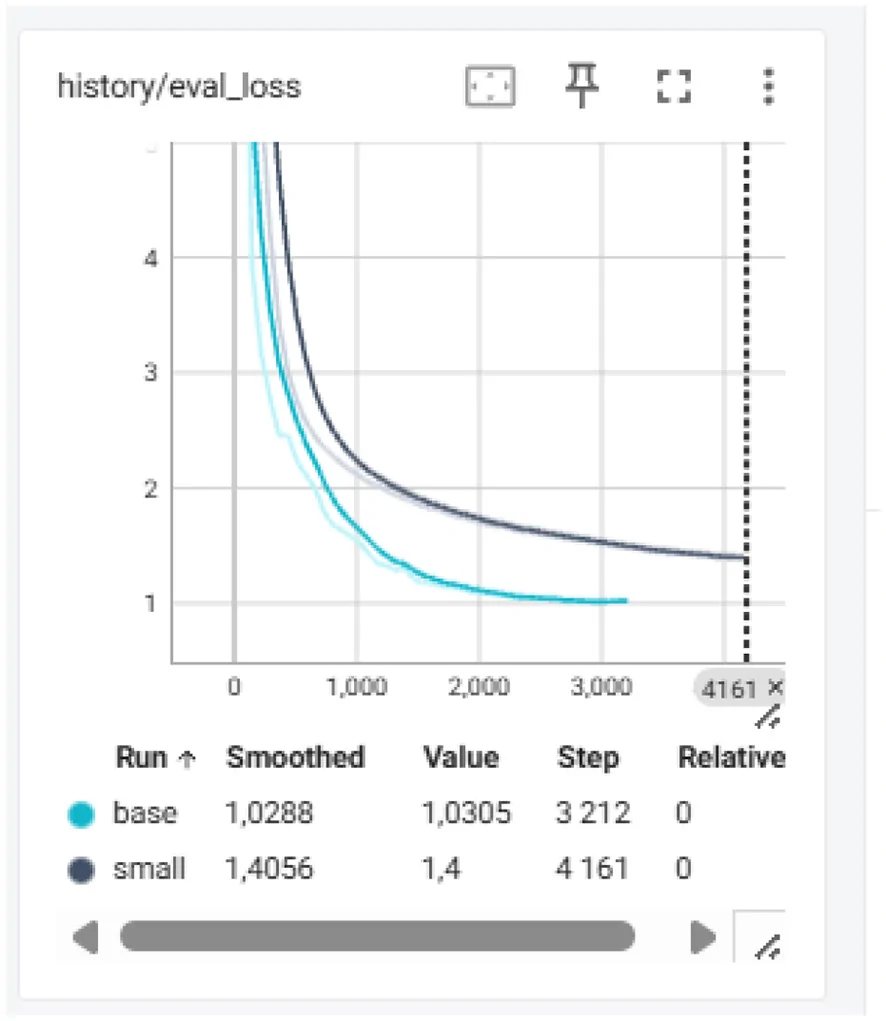
Training and validation loss during fine-tuning of mt5-small and mt5-base models.

**Figure 6:**
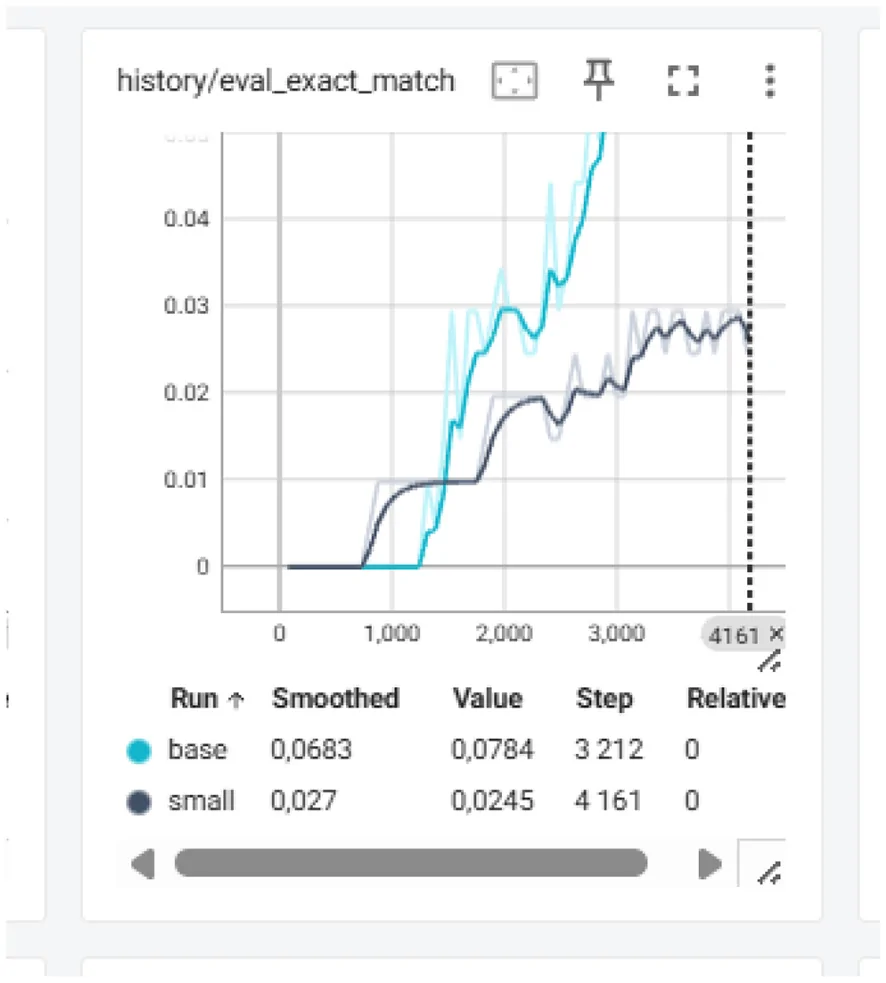
Evaluation metrics dynamics during training.

The dynamics of the loss function are shown in Figure 5. The mt5-base model shows faster convergence and lower validation error compared to the mt5-small model (see Figure 6).

The faster convergence of the mt5-base model can be attributed to its greater representational capacity, which allows more efficient modeling of complex morphological and syntactic dependencies of the Kazakh language, including the transformation of text structures into gloss sequences.

## 4 Results

Statistical indicators of the training process and generation quality metrics are presented in Tables 3, 4.

**Table 3:** Comparative characteristics of the learning process.

Model	Number of epochs	Early stopping	Optimizer	Eval loss
google/mt5-small	16	Yes	adamw	0.2564
google/mt5-base	40	Yes	adafactor	0.3474

**Table 4:** Gloss generation quality metrics.

Model	Accuracy (%)	BLEU	ROUGE-1 F1 (%)	ROUGE-L F1 (%)	Token-level coverage (%)	Exact match (%)
google/mt5-small	89.42	87.51	89.61	89.61	89.48	74.96
google/mt5-base	91.99	90.12	92.08	92.02	92.19	80.68

The interpretation of the metrics is based on several indicators. Accuracy reflects the positional accuracy of the tokens in the predicted sequence. The token-level coverage metric characterizes the ability of the model to reproduce the necessary lexical composition of the gloss sequence and, in the absence of a strict linear correspondence between the Kazakh text and glosses, it is an important indicator of semantic correspondence. The exact match measure determines the proportion of exact matches between the predicted and the reference sequences. The BLEU and ROUGE metrics are used to assess the quality of generation and reflect the similarity between the predicted and the reference sequences at the level of n-grams.

The results demonstrate the advantage of the google/mt5-base model in all evaluated metrics. In particular, there is an increase in BLEU (by 2.61 points, from 87.51 to 90.12) and ROUGE-1 F1 (by 2.47 points, from 89.61% to 92.08%), which may indicate more effective modeling of contextual dependencies.

Following the corpus expansion to 11,190 text–gloss pairs, exact match rose substantially for both models (74.96% for mT5-small, 80.68% for mT5-base), compared to below 6% on the earlier, smaller corpus. This confirms that exact-match performance in seq2seq gloss generation is strongly data-bound: the earlier low values primarily reflected data scarcity rather than an inherent ceiling for the task, and the model is able to reproduce reference sequences closely once sufficient parallel data is available.

Qualitative analysis of the results shows differences in the ability of the models to process complex structures. In particular, in Example 2, the mt5-base model more accurately reproduces numerical and terminological elements, including the tokens “нүкте” and “0,” while the mt5-small model partially omits them. This suggests that the mt5-base model is better able to preserve the structure of the statement when generating a gloss sequence.

To further characterize the nature of residual errors, we performed a token-level confusion analysis on the test set for both models presented in Table 5.

**Table 5:** Token-level error type breakdown on the test set.

Model	Substitutions	Omissions	Hallucinations
google/ mt5-small	47	11	2
google/ mt5-base	42	2	11

Substitution errors are dominated by morphologically or semantically close lexical variants (e.g., көтеру → көтерілу, шешім → бітім, ата → қарт) rather than unrelated confusions, indicating that residual errors are concentrated in fine-grained lexical disambiguation rather than structural mistakes. Inspection of the lowest-scoring predictions revealed no systematic word-order (reordering) errors, consistent with the largely monotonic nature of the text → gloss mapping in this corpus; we, therefore, categorize errors as substitution, omission, or hallucination. Notably, mT5-base hallucinates more often (11 vs. 2) but omits far less (2 vs. 11) than mT5-small, suggesting the larger model is more “confident” in always producing an output, at the cost of occasional spurious glosses.

## 5 Discussion

The results allow us to compare the effectiveness of the proposed approach with existing research in the field of sign language processing. The advantage of the mt5-base model over the mt5-small corresponds to trends in the use of transformers and transfer learning for TTG tasks, where increasing model capacity improves the extraction of contextual dependencies (Patil et al., 2022; Waghmare and Deshpande, 2024).

The substantial rise in exact-match accuracy observed after corpus expansion (see Results) reinforces prior observations that strict string-matching metrics are highly sensitive to training data volume in seq2seq tasks, and that low exact-match values at small data scales do not necessarily indicate poor semantic translation quality (Shahin and Ismail, 2024). Nonetheless, semantic interpretation of model outputs–beyond string-level metrics–remains important, since occasional residual errors (Table 5) still involve semantically close but non-identical lexical substitutions.

The structure and quality of the training corpus play an important role in the results. Studies, including work on morphologically complex languages (Sawalha et al., 2025), show that increasing annotation depth and data volume significantly improves translation quality. This study provides direct confirmation of this relationship: expanding the corpus from 1,942 to 11,190 pairs yielded substantial gains across all metrics (Tables 3, 4), underscoring the strong dependence of TTG model performance on the volume of parallel training data.

To situate our approach relative to other low-resource, morphologically rich languages, we highlight two methodological contrasts. Corpus construction for the Oran dialect of Arabic relied on fine-grained, morpheme-level annotation of roots and affixes (MADOran, 30,919 words) (Sawalha et al., 2025); we instead standardize glosses at the word/phrase level, which sacrifices some morphological granularity but made it feasible to annotate a substantially larger corpus (11,190 pairs). Separately, prior work on Urdu links persistently high MT error rates (~50%) to the absence of explicit interlinear glossing (Shah et al., 2024); our data support the same conclusion–exact-match accuracy improved sharply once the corpus was scaled up, confirming that gloss-level intermediate representations depend heavily on data volume in agglutinative languages, not only in Kazakh (Yerimbetova et al., 2025). Together, these comparisons point to a general trade-off between annotation granularity and achievable corpus scale in low-resource TTG systems.

A comparison with gloss-free SLT approaches shows that rejecting an intermediate representation can reduce the accuracy of reproducing morphological and structural features of sign language (Kim and Baek, 2023; Hwang et al., 2025). The results confirm that the use of glosses as an intermediate level is justified, especially for low-resource languages.

The limitations identified in pre-trained text model studies are confirmed in this study (De Coster and Dambre, 2022). Despite improvements in evaluation metrics, transformer models do not always account for the spatial-grammatical features of sign language, which explains the discrepancies between reference and generated sequences.

The results are consistent with current scientific findings and confirm that the key factors in improving the quality of TTG systems are the increase in the amount of annotated data, the improvement of glossing methods, and the adaptation of architectures to the features of sign languages.

## 6 Conclusion

Within the framework of this study, a corpus was developed and, on its basis, a dataset was constructed for the TTG task in Kazakh Sign Language. The generated resource includes 11 190 text–gloss pairs and is one of the first structured datasets for this language pair, intended for machine learning tasks.

Based on the dataset, two pre-trained transformer models were fine-tuned: mt5-small and mt5-base. Both models showed stable convergence and the ability to learn from limited data. The mt5-base model provided higher quality metrics, including improvements in BLEU, ROUGE, and token-level coverage.

The analysis showed that, following the corpus expansion, both models achieve high exact-match accuracy (74.96% for mT5-small, 80.68% for mT5-base), confirming that the models can reliably reproduce reference gloss sequences rather than merely generating semantically plausible but structurally divergent output. This represents a marked improvement over the earlier, smaller corpus, where exact-match values below 6% mainly reflected data scarcity rather than a ceiling on achievable translation quality.

The scientific novelty of this work lies in the development of a sizeable (11,190-pair), manually annotated, reusable corpus for Kazakh Sign Language–currently the only resource of its kind for this language–its formalization as a dataset for the TTG task, and the experimental comparison of the efficiency of transformer architectures under low-resource conditions. In addition, the comparison across two corpus sizes (1,942 vs. 11,190 pairs) provides direct empirical evidence that TTG exact-match performance is strongly data-bound, a finding likely relevant to other agglutinative, low-resource sign languages beyond Kazakh. Unlike existing research, the work focuses on an agglutinative language with limited resources, which emphasizes its practical significance.

The practical significance of the results lies in the use of the dataset and trained models in automatic translation systems, educational platforms, and assistive technologies for people with hearing impairments. In particular, the gloss sequences generated by the models described here serve as direct input to the SignBridge avatar animation pipeline, a bilingual Kazakh–Russian sign language avatar system validated through expert evaluation (mean score 5.5/7, Cronbach's α > 0.8) by certified sign language interpreters (Amangeldy et al., 2026), demonstrating an end-to-end path from text to rendered sign language animation.

Future research will focus on evaluating the end-to-end comprehensibility of the full text-to-sign-language pipeline–from text through gloss generation to avatar rendering–as a single integrated system, rather than assessing the TTG model and the avatar separately. This will involve a structured comprehensibility study conducted jointly with the Deaf community and certified Kazakh Sign Language interpreters, extending the expert-evaluation methodology already validated for the SignBridge avatar (Amangeldy et al., 2026) to directly assess whether end users correctly understand the meaning conveyed by model-generated gloss sequences once rendered as sign language animation. In parallel, further work will address expanding the corpus, improving annotation depth, and incorporating multimodal data (video and keypoints) to improve the completeness and naturalness of sign language generation.

## Data Availability

The original contributions presented in the study are included in the article/supplementary material, further inquiries can be directed to the corresponding author/s.
